# The pursuit of understanding human longevity

**DOI:** 10.1038/s41514-026-00339-z

**Published:** 2026-02-05

**Authors:** Pawel Kordowitzki, Kejun Ying

**Affiliations:** 1 Department of Basic and Preclinical Sciences, Nicolaus Copernicus University, Torun, Poland; 2 Department of Gynaecology, European Competence Centre for Ovarian Cancer, Charité Medical University, Berlin, Germany; 3 T. H. Chan School of Public Health, Harvard University, Boston, MA USA; 4 Wu Tsai Neurosciences Institute, Stanford University, Stanford, CA USA

**Keywords:** Evolution, Genetics, Physiology

## Abstract

Precise recommendations for humans to reach more than 100 years remain elusive. A recent multiomics study revealed that extreme age and poor health are not inherently linked. Longevity stems from a multifactorial resilience that involves protective genetics, efficient metabolism, low inflammation, and favorable lifestyle choices. Insights from centenarians and Blue Zones suggest that healthy aging is rooted in the synergistic interplay of biological, environmental, and above mentioned factors.

A groundbreaking study by Santos-Pujol and colleagues^1^ provides a comprehensive multiomics blueprint of the individual recognized as the world’s oldest validated living person from January 17, 2023, until her demise on August 19, 2024, having attained an age of 117 years and 168 days. Interestingly, their findings show that extreme advanced age and poor health are not intrinsically linked; people who live longest tend to be the healthiest. This profound decoupling suggests that the prescription for longevity isn’t about escaping aging entirely, but rather about maintaining remarkable resilience against age-associated diseases, meaning living a long time and remaining healthy until the end of life.

To formulate recommendations for a long and healthy life, a comprehensive approach integrating interdisciplinary insights from multiomics studies, centenarian populations, metabolic and anatomical research, animal models, and human epidemiology is essential. Each discipline offers a unique lens through which to understand the complex interplay of factors contributing to longevity. The multiomics study by Santos-Pujol and colleagues highlights the importance of a resilient genome, encompassing pro-longevity alleles associated with cardiovascular health, brain function, the immune system, and mitochondrial oxidative phosphorylation. Furthermore, dietary influences, such as yogurt intake, are suggested to contribute to a healthy gut microbiome, which in turn fosters healthy aging. In this regard, it is worth emphasizing that the utilization of *Bifidobacterium* as a probiotic intervention capable of attenuating the progression of numerous age-related disorders is garnering increasing interest^1^. The study also highlights metabolic interventions, such as calorie restriction and limiting specific nutrients like methionine. Diet, therefore, appears to play a crucial role in recommendations for a long and healthy life. Globally, there are certain regions, among others, the so-called “Blue Zones”, which have been well-investigated in this regard. These studies provide real-world examples of populations, such as those in Okinawa, Ikaria, Sardinia, Nicoya, and Loma Linda, that exhibit exceptional longevity. Common threads among these communities include primarily plant-based diets, regular physical activity integrated into daily life, strong social and family connections, and effective stress management. While their specific diets vary, the general pattern emphasizes whole, unprocessed, locally sourced foods^2^. Research indicates that metabolic pathways play a significant role in regulating aging, and metabolic reprogramming is a key driver of this process^3^. Targeting specific metabolic pathways has been shown to extend lifespan and healthspan across various species^3^. Critical pathways include insulin/IGF and mTOR, with caloric restriction recognized as a primary metabolic intervention. These findings underscore the pivotal role of cellular processes, including energy metabolism, DNA damage repair, nutrient sensing, protein homeostasis, and mitochondrial function, in regulating lifespan^4^.

Animal models provide controlled experimental evidence for the efficacy of specific dietary and lifestyle interventions. Calorie Restriction and Intermittent Fasting, for instance, consistently extend lifespan and healthspan in organisms ranging from yeast to rodents and monkeys. These interventions modulate key nutrient-sensing pathways, including AKT, FOXO, mTOR, NAD + , AMPK, and FGF21, thereby reinforcing the metabolic link to longevity^5^. Ultimately, human epidemiology studies provide population-level evidence, consistently showing that maintaining a healthy weight, prioritizing the quality of food types over quantity, and adhering to specific healthy dietary patterns are crucial for longevity. Diets like the Mediterranean, Nordic, Okinawa, DASH, and healthy plant-based diets are strongly associated with reduced mortality and healthy aging^6^. These patterns typically feature an abundance of fruits, vegetables, whole grains, nuts, legumes, and lean proteins, while limiting red and processed meats, trans fats, sodium, and sugary beverages. Lifestyle factors, such as avoiding tobacco and excessive alcohol, also significantly reduce the risk of age-associated chronic diseases. By integrating these multifaceted findings, we can develop holistic, evidence-based recommendations that address both the molecular underpinnings and the observable lifestyle patterns associated with healthy aging and longevity.

The Santos-Pujol study highlights why and how we value healthy aging. The high prominence of aging as the primary risk factor for life-threatening diseases, such as cancer, cardiovascular diseases, and neurodegenerative disorders, has significantly propelled the pursuit of longevity in the last decades; however, the prescription for humans reaching more than 100 years still requires further elucidation. Aging, in biological terms, is defined as a decline in function and a reduction in the ability to heal from injuries, infections, and diseases, as well as an increased risk of contracting illnesses and mortality over time^7^. Importantly, the study of Santos-Pujol and colleagues^1^ elucidates that longevity is not governed by a single biological switch, but rather a combination of robust protective mechanisms. These include advantageous genetic variants offering neuroprotection, efficient mitochondrial function, and cardioprotection. Crucially, the supercentenarian exhibited low levels of inflammation, efficient lipid metabolism, a rejuvenated gut microbiome, and a CpG methylation status resembling that of younger individuals.

A potential plausible explanation for why centenarians may experience more favorable inflammation outcomes lies in their distinctive genetic background, particularly the expression patterns of longevity- and vulnerability-related genes^8–10^. Noteworthy, in extreme old age, IL-6 has been proposed to exert a pivotal influence on the extent of morbidity and mortality. A study involving Chinese centenarians reported a significant association between the IL6 polymorphism rs2069837 and exceptional longevity^4^. In this context, recent work has provided direct genomic evidence that centenarians are genetically protected not only by advantageous variants but also by the absence of damaging ones. Specifically, Ying et al.^11^ demonstrated that individuals achieving exceptional longevity exhibit a significant depletion of rare loss-of-function mutations across the genome, suggesting that reduced deleterious burden contributes to resilience and health span. This study identified multiple candidate longevity genes with causal effects on aging-related traits, providing a mechanistic foundation for the protective genetic architecture observed in long-lived individuals. Interestingly, among the top-ranked transcription factors driving the distinctive gene expression profile of M116 stand out potent modulators of immune and inflammatory responses, including the extensively studied nuclear factor κB/RELA (RelA; avian reticuloendotheliosis viral oncogene homolog A) network, the critical MHC regulator RFX5, and the pivotal STAT1/STAT2 complex that robustly governs interferon-stimulated gene expression^1^. These unique expression profiles may underpin an enhanced immune capacity to counter infectious diseases.

Importantly, the recent study by Santos-Pujol et al^1^. also acknowledges the limitations of drawing broad conclusions from a single centenarian. It points towards diverse intervention targets for delaying aging and enhancing health span, ranging from dietary influences and exercise to metabolic tuning, and potentially drugs that protect telomeres or modulate epigenetics^12,13^. Future research must build upon these multiomics insights with larger cohorts and longitudinal studies to translate these discoveries into actionable strategies for healthier, longer lives for all. In this context, it is worth noting that the exceptional longevity observed in the world’s so-called Blue Zones is not attributable to a single cause, but rather arises from a complex convergence of environmental, lifestyle, and potentially genetic factors^14–16^. Besides, physical activity constitutes a fundamental element of a healthy lifestyle, exhibiting a well-established dose-response relationship with longevity^16–18^. It has been shown that centenarians do not prioritize strenuous exercise; rather, physical activity is intrinsically woven into their daily lives through practices such as walking, gardening, and traditional manual labor^19^. This sustained, low-intensity engagement fosters cardiovascular health, preserves muscle mass, and supports metabolic function^20^. Regular physical activity yields numerous health benefits, including enhanced cardiovascular function and improved psychological well-being^21,22^. The profound significance of robust social connections and community participation within centenarian populations cannot be overstated. Across these populations, individuals maintain deep ties with family, friends, and their wider community, cultivating a strong sense of belonging and mutual support^23^. These extensive social networks offer emotional resilience, mitigate stress, and promote mental health, all of which contribute to extended lifespans. Social engagement is recognized as a critical determinant of active aging^24^. Consequently, maintaining an active social life is associated with increased longevity.

In summary, this Comment article aims to highlight that exceptional human longevity arises not merely from postponed aging, but from the concurrent presence of two biologically disparate and ostensibly contradictory conditions that can occur in one individual. Long-lived individuals exhibit canonical indicators of advanced age, such as telomere attrition, accumulation of clonal hematopoiesis-related mutations, and age-associated shifts in immune cell profiles, including senescent B-cell characteristics, features conventionally linked to heightened frailty and physiological deterioration. Strikingly, despite these aging signatures, centenarians concurrently retain multiple safeguarding molecular and functional attributes, encompassing genetic polymorphisms protective against prevalent age-related pathologies, a metabolically advantageous lipidomic profile, an anti-inflammatory gut microbiota composition, and an epigenomic profile characterized by genomic integrity and attenuated epigenetic clocks. The originality of the Santos-Pujol observations resides in delineating this dichotomous biological paradigm, wherein senescent biomarkers coexist with sustained functional safeguards. This paradigm challenges the conventional notion of longevity as solely attributable to decelerated aging and proposes an alternative framework in which resilience, plasticity, and targeted preservation of critical molecular cascades are pivotal to achieving a protracted lifespan and healthspan.

## Data Availability

No datasets were generated or analysed during the current study.
